# A mixed methods randomised feasibility trial investigating the management of benign paroxysmal positional vertigo in acute traumatic brain injury

**DOI:** 10.1186/s40814-020-00669-z

**Published:** 2020-09-16

**Authors:** Rebecca M. Smith, Natalie Marroney, Jenna Beattie, Abby Newdick, Vassilios Tahtis, Caroline Burgess, Jonathan Marsden, Barry M. Seemungal

**Affiliations:** 1 Brain And Vestibular Group (BAVG), Neuro-otology Unit, Department of Brain Sciences, Imperial College London, London, UK; 2 Imperial College Healthcare NHS Trust, London, UK; 3 St George’s University Hospitals NHS Foundation Trust, London, UK; 4 King’s College Hospital NHS Foundation Trust, London, UK; 5 Population of Health Sciences, King’s College London, London, UK; 6 School of Health Professions, University of Plymouth, Plymouth, UK

**Keywords:** Feasibility study, Traumatic brain injury, Benign paroxysmal positional vertigo, Rehabilitation

## Abstract

**Background:**

Traumatic brain injury (TBI) is the leading cause of long-term disability in working age adults. Recent studies show that most acute TBI patients demonstrate vestibular features of dizziness and imbalance, often from combined peripheral and central vestibular dysfunction. Effective treatment for vestibular impairments post-TBI is important given its significant adverse impact upon quality of life and employment prospects. The most frequent peripheral vestibular disorder in acute TBI is benign paroxysmal positional vertigo (BPPV), affecting approximately half of acute cases. Although there is effective treatment for idiopathic BPPV, there are no high-quality clinical data for post-TBI BPPV regarding its prevalence, natural history, which treatment is most effective and when is the best time to treat. In particular, observational studies suggest post-TBI BPPV may be recurrent, indicating that hyperacute treatment of BPPV may be futile. Given the potential hurdles and the lack of accurate post-TBI BPPV data, the current study was designed to provide information regarding the feasibility and optimal design of future large-scale prospective treatment studies that would compare different interventions and their timing for post-TBI BPPV.

**Method:**

A multi-centre randomised mixed methods feasibility study design was employed. We aim to recruit approximately 75 acute TBI patients across a range of clinical severities, from three major trauma centres in London. Patients will be randomised to one of three treatment arms: (1) therapist-led manoeuvres, (2) patient-led exercises and (3) advice. Participants will be re-assessed by blinded outcome assessors at 4 and 12 weeks. Acceptability of the intervention will be obtained by patient interviews at the end of their treatment and therapist interviews at the end of the study. Primary outcomes relate to feasibility parameters including recruitment and retention rates, adverse events and intervention fidelity. We will also aim to provide a more accurate estimate of the prevalence of BPPV in TBI cases on the trauma ward.

**Discussion:**

The multi-centre nature of our feasibility study will inform the design of a future prospective treatment trial of BPPV in acute TBI. Important parameters we will obtain from this study, key for designing a future prospective treatment study, include estimating the prevalence of BPPV in TBI patients admitted to UK major trauma wards, and elucidating both patient and care-provider barriers in delivering BPPV treatment.

**Trial registration:**

ISRCTN, ISRCTN91943864. Registered on 10 February 2020.

## Background

Traumatic brain injury (TBI) is the most common cause of long-term disability in working age adults [1]. Indeed, in England and Wales, it is estimated TBI results in 1.4 million emergency visits per year [2], costing the economy £15 billion [3] and resulting in a significant impact on health-related quality of life for patients and carers [4]. Amongst the plethora of impairments experienced by TBI survivors, vestibular features or complaints of dizziness and/or imbalance are common, with large numbers of patients remaining symptomatic at 5-year follow-up [5].

The impact of dizziness on TBI survivors can be varied and burdensome, affecting physical, psychological and socioeconomic domains [6]. Acute and long-lasting dizziness can directly affect physical health, with implicit links to imbalance and therefore risk of falls [7, 8], which affect half of community-dwelling TBI survivors [9, 10]. The well-documented impact of falls on physical and psychological morbidity, mortality [11] and healthcare resource consumption [12] necessitates risk factors such as dizziness be appropriately managed. Imbalance and dizziness can also impair mental wellbeing, as demonstrated by studies describing direct links between the vestibular system and brain areas involved in emotional and cognitive processing [13–15]. Further, Chamelian et al. (2004) demonstrated dizziness to be an independent predictor of return to work, reporting only a third of dizzy patients had returned to work at 6 months compared to three quarters of patients without dizziness [16]. The implications of long-term unemployment not only include a socioeconomic burden for patients and their families, but also involve a wider impact on work productivity and costs for employers and society. Indeed, findings from a large observational study demonstrated the majority of patients with dizziness had reduced their workload and lost working days at just 3 months post onset of symptoms [17]. Therefore, the complex interplay between dizziness, physical health, neuropsychiatric function and socioeconomic factors provides the rationale for enhancing early diagnosis and treatment of dizziness.

**Fig. 1:**
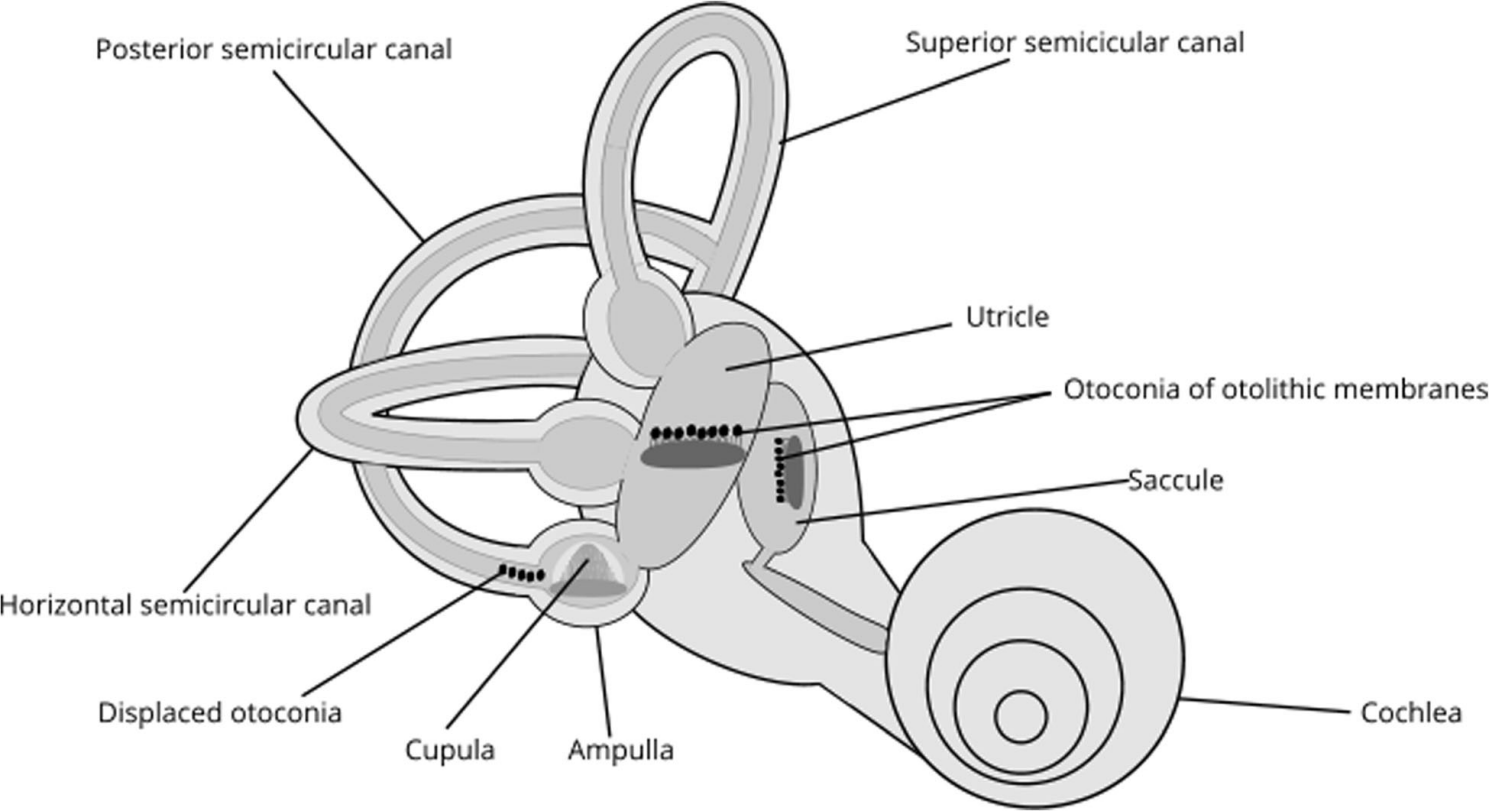
Schematic representation of displaced otoconia within the posterior semi-circular canal. Figure kindly provided by John Corcoran, April 2020

The most frequently diagnosed cause of dizziness following TBI is benign paroxysmal positional vertigo (BPPV), theorised to be due to mechanical displacement of calcium carbonate ‘crystals’ or otoconia (Fig. 1) during the head injury itself [18]. As a result, patients typically, but not always, experience a sense of illusory self-motion when moving their head. Importantly, idiopathic BPPV is also associated with imbalance and falls [19] and hence, BPPV could exacerbate imbalance due to brain injury. Surprisingly, there is little evidence exploring falls in acute post-traumatic BPPV. Previous research in sub-acute TBI demonstrates the prevalence of post traumatic BPPV to range from 11 to 28% [20–22]; however, pilot data collected in acute TBI patients at St Mary’s Hospital, London, suggests it may be as high as 55% [23]. Further work is indicated to establish more accurate prevalence data. In particular, post-traumatic BPPV is thought to be more complex than the idiopathic form, with higher rates of multi-canal diagnoses and more treatment sessions required for resolution [21]. However, conflicting data exists regarding recurrence rates [21, 24].

Robust clinical guidelines [25] direct the treatment of idiopathic BPPV, involving repositioning manoeuvres or repeated movements of the head and body to relocate the otoconia within the inner ear canals. However, there is a relative paucity of research regarding treatment of post traumatic BPPV, in particular regarding factors such as optimum dosage, or the most beneficial time to treat. The few studies which have examined treatment effectiveness were solely completed in sub-acute or chronic TBI patients and are further limited by their retrospective nature [21, 24], small sample size [20, 21] and lack of focus on BPPV resolution, symptom burden, or other patient-centred outcomes. The scarcity of acute prospective data noted may be a factor in the variability noted in routine assessment and treatment of post traumatic BPPV.

Indeed, despite the relatively high prevalence of post traumatic BPPV, emerging evidence suggests patients are not routinely assessed during the acute stages of their injury [26]. Our pilot work corroborates such data, indicating variable assessment and treatment of post traumatic BPPV appears to be complicated by two factors: (1) vestibular agnosia—impaired dizziness sensation which is also linked to imbalance via damage to the right temporal lobe circuits [27], and (2) clinician factors including insufficient training or skills. Vestibular agnosia may lead to a reduction in patients’ ability to report dizziness. This is clinically meaningful as patients with treatable diagnoses such as BPPV will not be assessed or treated if screening is solely based on symptoms. An initial report by Calzolari et al. [27] demonstrated vestibular agnosia leads to a seven fold reduction in clinician recognition of BPPV [27]. Furthermore, early results from a qualitative study exploring barriers to vestibular assessment and treatment at a major trauma centre in London demonstrated a variety of role and knowledge-based factors were linked with a lack of routine assessment and treatment of dizziness [28]. This is perhaps unsurprising given national head injury guidelines contain no reference to ‘vestibular’ or ‘dizziness’ assessments following head injury [2]. Therefore, the presence of vestibular agnosia and the lack of routine vestibular assessment and treatment may heighten the risk of clinically significant conditions such as post traumatic BPPV remaining undiagnosed, with patients predisposed to the acute and long-lasting sequelae of dizziness.

In summary, BPPV seems to affect the majority of acute TBI patients with possible long-lasting physical, psychosocial and economic consequences. Despite this, for a variety of reasons acute assessment and treatment is sub-optimal, whilst insufficient data exists to support decisions regarding effective treatment.

### Aim and objectives

The primary aim of this study is to evaluate the feasibility of conducting a future randomised controlled trial (RCT) investigating the effectiveness of treating BPPV in acute TBI.

Primary objectives focus on feasibility outcomes and will aid the design of a future definitive trial:

- To establish trial recruitment rates, including the willingness of patients to be randomised to a treatment arm
- To determine trial retention rates, the fidelity of the intervention, its acceptability to participants and clinicians and any missing data
- To explore the incidence of adverse events

Secondary objectives will also support the design of a future RCT. They include:

- An estimate of the prevalence of BPPV in acute TBI
- Calculating effect sizes for primary and secondary outcome measures to thereby inform sample size estimates for future studies
- Examining the relationship between vestibular perception, balance impairment and clinical outcome

## Methods

### Study design

Currently, it is not routine practice in all major trauma centres to assess for or treat BPPV in acute TBI. In order to implement any long-term changes to screening and/or treatment behaviours, it is necessary to identify any possible barriers to such changes in clinical practice. Therefore, a feasibility study utilising both quantitative and qualitative methods was deemed appropriate.

**Fig. 2:**
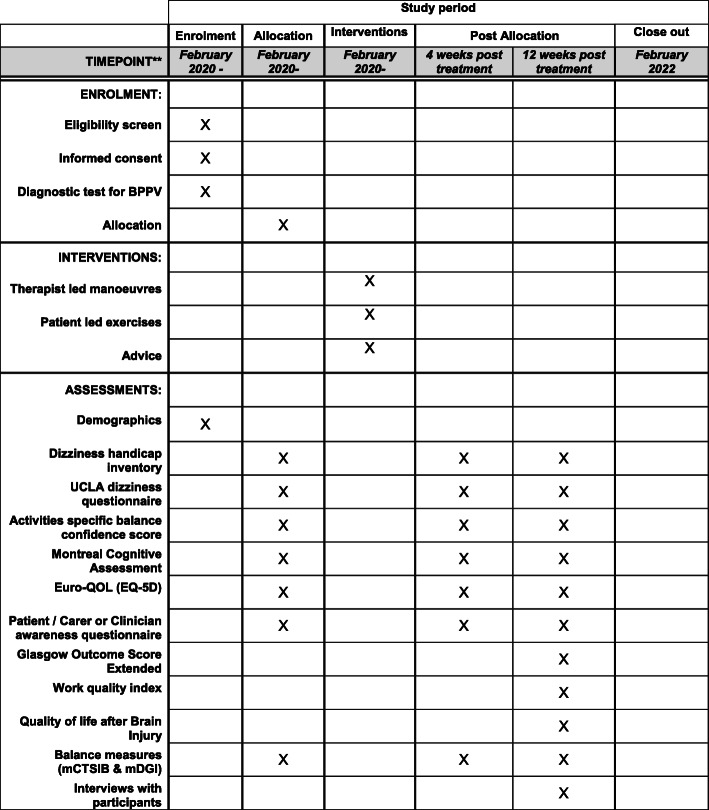
SPIRIT figure

This multi-centre, mixed methods randomised feasibility trial will comprise a randomised trial with two intervention arms and one comparator arm, and a subsequent qualitative study (Fig. 2). Reporting of this protocol will comply with the SPIRIT statement.

- Phase 1 will consist of a feasibility trial involving approximately 75 patients randomised to one of the three arms.
- Phase 2 will qualitatively explore the experiences of patients and clinicians participating in the trial and their views regarding progression to a randomised controlled trial. Patients who decline to take part will be invited for their feedback to enhance recruitment for a future trial.

### Phase 1—feasibility trial

#### Recruitment

Trauma care in England is delivered through specialist centres [29]. Indeed, patients will be recruited from major trauma centres in London. Direct members of the care team will screen patients using inclusion and exclusion criteria and provide eligible patients with an information sheet and consent form.

#### Eligibility criteria

Inclusion criteria consists of patients over the age of 18 with a non-penetrating head injury, able to provide informed consent (or with a consultee willing to provide advice), inpatient on a trauma or outlying ward and a sufficient grasp of English to complete outcome measures. Patients will be excluded if they have a current history of substance abuse, are medically unstable, are pregnant, have vascular or orthopaedic injuries precluding neck extension, have active psychiatric disease or a history of psychotic disease, have a Glasgow Coma Score less than 14, or have a current prescription of phenytoin.

#### Assessment

**Fig. 3:**
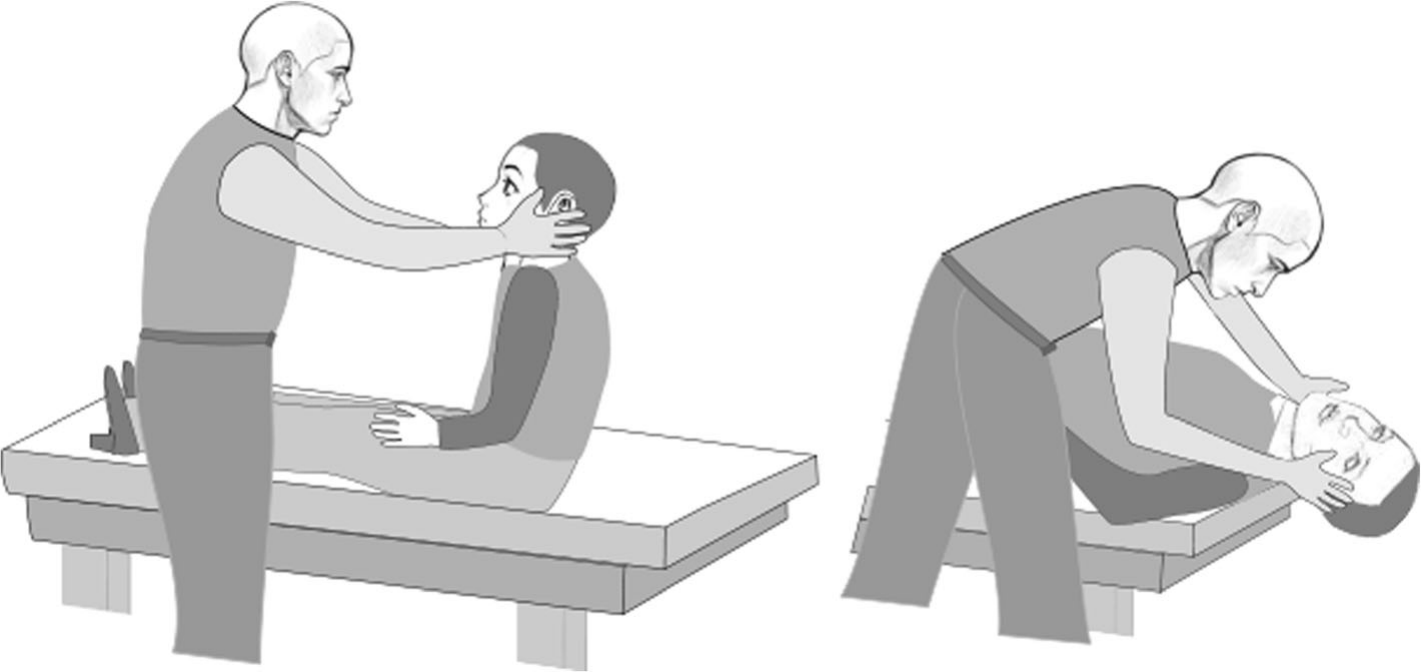
Schematic representation of the Dix Hallpike, the diagnostic test for BPPV. Patients lie supine with their head rotated 45° and extended 20°. Figure kindly provided by John Corcoran, April 2020

Eligible patients who consent to take part in the study will be assessed by a ward therapist for BPPV using a Dix Hallpike manoeuvre (Fig. 3), which is the gold standard diagnostic test [25]. Therapists (occupational therapists and physiotherapists) will be trained to undertake such manoeuvres prior to the study commencing.

#### Randomisation

**Fig. 4:**
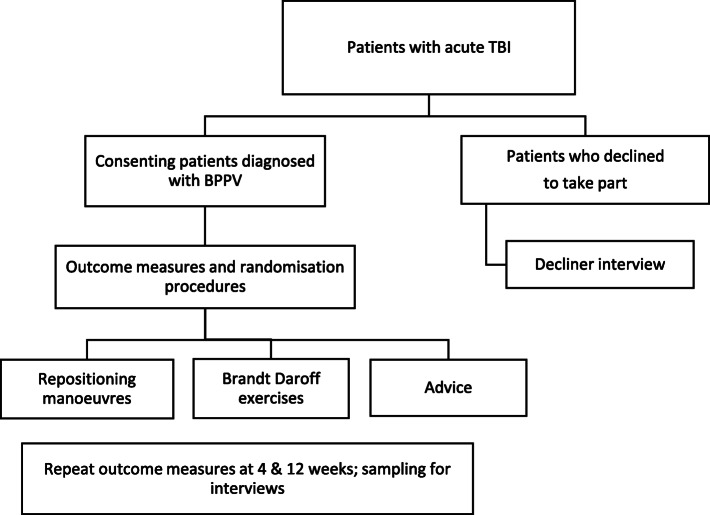
Simplified study flow chart

Those diagnosed with BPPV will be randomised to one of three intervention arms (Fig. 4). Patients testing negative for BPPV will not take further part in the trial. Randomisation will occur through an online randomisation platform (Sealed Envelope) which uses sequential treatment assignment. Minimisation criteria will be used to ensure groups are balanced. Trial participants and outcome assessors will be blinded to group allocation; however, due to the physical nature of the interventions, blinding of the therapists delivering the intervention will not be possible.

#### Interventions

Participants will be randomly allocated to one of three arms: (1) therapist-led repositioning manoeuvres, (2) patient-led exercises, or (3) advice.

1. *Therapist-led repositioning manoeuvres*

Repositioning manoeuvres are physical movements of the head and body typically used to treat idiopathic or traumatic BPPV [25]. Ward therapists will be taught to perform repositioning manoeuvres as per clinical guidelines [25]. Those diagnosed with BPPV affecting both left and right sides will be treated with the most symptomatic side first (or the side with the most intense nystagmus if the participant is not symptomatic). A repeat diagnostic test will be subsequently performed to determine the success of the intervention. The number of manoeuvres required for resolution of BPPV will vary between participants. If acceptable, the therapist will continue completing manoeuvres, up to a maximum of three manoeuvres per session, and a maximum of three sessions (over subsequent days where applicable). Participant discomfort, nausea, or vomiting would be indications to cease treatment. Any adverse events or reasons to stop the intervention will be recorded.

2. *Patient-led exercises*

Patient-led or Brandt-Daroff exercises are a form of physical self-treatment involving repeated movements of the head and trunk prescribed by clinicians as an alternative to, or in tandem with, repositioning manoeuvres. The National Institute of Clinical Evidence suggest Brandt-Daroff exercises as a treatment option for idiopathic BPPV [30], although their effectiveness is unknown post TBI.

Ward therapists will teach participants and carers (where required) the Brandt-Daroff exercises. A repeat diagnostic test will be completed. Following treatment, participants will be instructed to complete the exercises twice per day for 2 weeks, using a standardised, written exercise sheet. Participants will be asked to complete a home diary to record their adherence with the exercises.

3. *Advice*

Ward therapists will provide participants with verbal and written advice. This will comprise ensuring adequate fluid intake, taking precautions when mobilising independently on the ward or at home and trying to ensure normal head and body movement patterns. Advice will be provided in the course of the participant’s hospital stay, during a scheduled therapy session. Such advice is based on evidence suggesting dizzy patients who avoid head movements may be prone to motion sensitivity, which can delay recovery and contribute to long-lasting subjective dizziness and imbalance [31, 32]. A brief further session, repeating the advice will be provided prior to participant’s discharge. Following the last treatment session, a repeat diagnostic test will be performed to ascertain the presence of BPPV.

#### Intervention fidelity

Fidelity monitoring is an important part of any rehabilitation research intervention, enabling investigators to differentiate between comparator and experimental interventions and ensuring any procedures are carried out as defined in the protocol or guidelines [33]. In this study, video will be used to record therapists performing the interventions. Independent raters will observe and evaluate approximately 10% of therapy sessions, using a competency record sheet. This approach has been previously utilised in studies evaluating treatment fidelity [33].

#### Outcome measures

Baseline demographic data will be recorded including age, gender, factors relating to the head injury, functional status, falls and past medical history. Primary outcome measures relate to feasibility objectives: number of eligible patients, time taken to fulfil recruitment target, trial dropout rates, variability in intervention fidelity, proportion of outcome measures completed and any adverse events noted throughout the trial.

Secondary outcomes will be collected at three timepoints:

- Timepoint 0—during hospital stay, following consent, randomisation and prior to treatment
- Timepoint 1—at 4 weeks following treatment
- Timepoint 2—at 12 weeks following treatment

Secondary outcomes comprise the diagnostic test for BPPV (Dix Hallpike manoeuvre) to explore the frequency and recurrence rate of BPPV, in addition to a range of self-reported measures explored below.

Self-reported disability pertaining to dizziness will be evaluated using the Dizziness Handicap Inventory (DHI), a 25-item questionnaire with high test re-test reliability and low error of measurement score [34]. Dizziness frequency and severity will be examined by the UCLA Dizziness questionnaire [35]. The Activities-specific Balance Confidence scale will explore subjective balance confidence by asking participants to rate how balanced they feel completing sixteen different day to day tasks. Previous research in older adults has demonstrated scores less than 67% indicate a high risk of falling [36].

Cognitive function will be screened using the Montreal Cognitive Assessment (MoCA), a standardised test of visuo-spatial and executive function, naming, memory, attention, language, abstraction and orientation, routinely used in clinical settings [37]. Health outcome will be measured using the EQ-5D, a widely used tool consisting of five items regarding mobility, usual care activities, self-care, anxiety and depression and pain. Scores will also be used for economic evaluation during a definitive trial [38]. Anxiety and depression will be screened using the Hospital Anxiety and Depression scale (HADS). Participants respond by rating each question on a 4-point scale, with higher scores indicating increased levels of anxiety and/or depression [39].

Three further questionnaires will be used at timepoints 1 and 2 to explore functional recovery, return to work and quality of life. The Glasgow Coma Outcome Score is a tool extensively used in phase III trials as a primary measure of recovery following TBI [40]. The extended version of the measure, via structured interview, splits the latter three of five categories (death, vegetative state, severe disability, moderate disability and good recovery) into upper and lower domains to improve sensitivity. Return to work following TBI will be measured using the Work Quality Index—a measure of both work status and stability [41]. Participants are divided into four working status categories, and then subsequently into four working stability domains, giving a composite measure of work quality. The Quality of Life after Brain Injury measure was specifically developed for the TBI population and has been validated through two large multi-centre studies [42]. Participants rate their quality of life on 37 items in six scales assessing areas of cognition, self, daily life and autonomy, social relationships and their emotions and physical problems.

Objective balance outcomes (modified clinical test of sensory integration in balance (mCTSIB) and modified Dynamic Gait Index (mDGI)) will be measured at all three time points. The mCTSIB evaluates how participants are able to use sensory inputs during static balance in four conditions; feet together and eyes open or closed on a hard or soft surface for 30 s. This test has demonstrated excellent reliability and validity in those with vestibular disorders [43]. The mDGI comprises eight dynamic walking tasks. Raters evaluate gait pattern, level of assistance and time taken to complete the task. The psychometric properties of this measure have been investigated in several neurological populations including TBI [44, 45].

#### Sample size

As this is a feasibility study, no formal sample size calculation has been undertaken. However, Sim and Lewis [46] recommend at least 50 participants for this type of study. Accordingly, approximately 25 participants will be recruited into each arm, giving a total sample size of 75 patients. Our sample size compares favourably to previous trials investigating treatment of idiopathic BPPV [47, 48] and other feasibility studies [49, 50]. We will use secondary outcome measures to inform a sample size calculation for a future RCT.

A sample of up to ten patients who declined to take part in the study will be interviewed to examine their reasons for declining and therefore optimise recruitment strategies for a future study. Approximately ten therapists and 18 patients participating in the trial will also be interviewed at the end of the trial to explore intervention acceptability. Sample sizes for the qualitative component were drawn up following consultation of relevant guidance [51] which advocates an emphasis on sampling diversity as well as size in feasibility studies.

### Phase 2—interviews with participants and therapists

The aim of this phase will be to explore the feasibility of the trial from a participant and therapist perspective using face to face, individual interviews. A topic guide will be used to structure the interviews and will examine participants’ and therapists’ views on the study design and their experience of recruitment processes, assessments, outcome measures, the intervention itself and follow-up assessments. Data from this phase will be utilised to refine the study procedures to maximise recruitment for a subsequent RCT.

Potential participant interviewees will be purposively sampled at the 12-week follow-up timepoint. A sample of approximately 18 participants (6 from each treatment arm across all centres) will explore the thoughts and views of those taking part in the trial. Additionally, up to 10 participants who declined to participate in the study will be invited to take part in individual interviews to explore reasons for non-participation. They will be interviewed face to face or by telephone, according to preference. Up to ten therapists participating in the trial across all centres will be asked to consent to individual interviews.

#### Planned data analysis

Results will be reported descriptively to chart demographic and observational data. Participant recruitment/retention data will be reported using pilot and feasibility CONSORT guidelines [52]. An estimate of the prevalence of BPPV in acute TBI will be reported with 95% confidence intervals. The analysis will be undertaken on an ‘intention to treat’ basis, i.e. analysis will include all patients at the end of the study, whether or not they complete the intervention. We will assess the predictors of (i) return to work and (ii) BPPV at 3 months via a binary multiple logistic regression with predictors: DHI score, intervention group, duration of post traumatic amnesia and the presence of skull fracture. Our projected sample size of 75 patients should be sufficient to test these regression models given that each one has four variables. As there are two regression models, which both use the same predictor variables, the alpha will be adjusted (*α* = .025) based on the Bonferroni correction. We do not expect to demonstrate significant results from this data; rather, it will be used to calculate effect sizes for future prospective treatment studies.

Qualitative data will be transcribed verbatim and analysed using a Framework approach [53, 54]. This type of analysis uses a series of interconnected stages which enables the researcher to move back and forth across the data until a coherent account emerges. Following familiarisation with the transcripts, a framework is developed according to key issues or themes, into which data is then indexed. Data is then summarised by category and charted using the final coding index. Finally, key characteristics and differences between data are identified, allowing the generation of concepts, connections and relationships.

#### Study monitoring and data protection

A steering group comprising of an external chair, statistician, lay members and ward therapists from all sites discussed and provided input into the protocol at the planning stage. The steering group will meet throughout the duration of the trial to monitor recruitment and any adverse events. Additionally, a data monitoring committee will have access to unblinded data and will therefore be able to oversee the safety of the trial participants. Trial cessation criteria include any patient safety concerns, the futility of treatment in a specific intervention arm and recruitment of 75 patients.

Written data will be stored securely at individual NHS centres. Study data will be collected and managed using REDcap electronic data capture tools hosted at Imperial College London [55, 56]. REDcap (Research Electronic Data Capture) is a secure, web-based software platform designed to support data capture for research studies.

#### Trial progression criteria

Trial progression criteria will be used to determine whether it is appropriate to move forwards to a RCT. Criteria include:

- 60% of those screened eligible for inclusion.
- Initially 30% of eligible patients are expected to consent to participate in the study, rising to 50% of those eligible consented as the research team gain confidence with consenting procedures.
- ≤ 40% drop out rate.

Thresholds for participants consenting have been informed from previous feasibility studies in acute TBI [57, 58] and reflect some of the challenges of recruiting acute TBI patients [59].

#### Patient and public involvement

This feasibility trial was designed following discussions with patients from a specific brain injury charity. Patient members form an important component of the steering group. Feedback from patient members was invited on the proposed trial design, protocol and participant facing documents, such as information sheets and topic guides for interviews. Patients and the public will play a central role in planning dissemination of results and future steps.

## Discussion

This paper describes the rationale and the methods of a mixed methods feasibility trial for managing BPPV in acute TBI. The results of this feasibility trial will not only inform the design and development of a future RCT, but will also highlight and describe the prevalence of BPPV in this population. Long-term implications of diagnosing and treating BPPV early on in acute TBI may have a positive impact on patient centric outcomes including reduced symptom load, attenuated falls risk and a shortened delay in return to work. Additional service benefits include a reduced hospital length of stay, improved rehabilitation outcome and a lessened burden on outpatient resources following discharge.

## Data Availability

Not applicable.

## Abbreviations

TBI: Traumatic brain injury
BPPV: Benign paroxysmal positional vertigo
RCT: Randomised controlled trial

## References

[1] Langlois J, Rutland-Brown W, Thomas K (2006). The epidemiology and impact of traumatic brain injury: a brief overview. J Head Trauma Rehabil.

[2] Hodgkinson S, Pollit V, Sharpin C, Lecky F (2014). Early management of head injury: summary of updated NICE guidance. BMJ..

[3] Tennant A. Acquired Brain Injury: The numbers behind the hidden disability [Internet]. Headway, UK. 2015 [cited 2018 Feb 9]. p. 1–12.

[4] Vogler J, Klein A-M, Bender A (2014). Long-term health-related quality-of-life in patients with acquired brain injury and their caregivers. Brain Inj.

[5] Berman J, Fredrickson J (1978). Vertigo after head injury - a five year follow up. J Otolaryngol.

[6] Kleffelgaard I, Langhammer B, Hellstrom T, Sandhaug M, Tamber AL, Soberg HL (2017). Dizziness-related disability following mild–moderate traumatic brain injury. Brain Inj.

[7] Agrawal Y, Carey J, Della Santina C, Schubert M, Minor L (2009). Disorders of balance and vestibular function in us adults: data from the national health and nutrition examination survey, 2001-2004. Arch Intern Med.

[8] Deandrea S, Lucenteforte E, Bravi F, Foschi R, La Vecchia C, Negri E (2010). Risk factors for falls in community-dwelling older people: a systematic review and meta-analysis. Epidemiology..

[9] McKechnie D, Pryor J, Fisher MJ (2015). Falls and fallers in traumatic brain injury (TBI) rehabilitation settings: an integrative review. Disabil Rehabil.

[10] Murphy MP, Carmine H, Kolakowsky-Hayner S (2014). Modifiable and nonmodifiable risk factors for falls after traumatic brain injury: an exploratory investigation with implications for medication use. Rehabil Nurs.

[11] Ghajar J (2000). Traumatic brain injury. Lancet..

[12] Hartholt K, van Beeck E, MD P, Polinder S, van der Velde N, MD P, et al. (2011). Societal consequences of falls in the older population: injuries, healthcare costs, and long-term reduced quality of life. J Trauma.

[13] Indovina I, Riccelli R, Chiarella G, Petrolo C, Augimeri A, Giofrè L (2015). Role of the insula and vestibular system in patients with chronic subjective dizziness: an fMRI study using sound-evoked vestibular stimulation. Front Behav Neurosci.

[14] Indovina I, Riccelli R, Staab JP, Lacquaniti F, Passamonti L (2018). Personality traits modulate subcortical and cortical vestibular and anxiety responses to sound-evoked otolithic receptor stimulation. J Psychosom Res.

[15] Riccelli R, Indovina I, Staab JP, Nigro S, Augimeri A, Lacquaniti F (2017). Neuroticism modulates brain visuo-vestibular and anxiety systems during a virtual rollercoaster task. Hum Brain Mapp.

[16] Chamelian L, Feinstein A (2018). Outcome after mild to moderate traumatic brain injury: the role of dizziness. Arch Phys Med Rehabil.

[17] Benecke H, Agus S, Kuessner D, Goodall G, Strupp M (2013). The burden and impact of vertigo: findings from the REVERT patient registry. Front Neurol.

[18] Fife TD, Von Brevern M (2015). Emergency neuro-otology: diagnosis and management of acute dizzienss and vertigo.

[19] Oghalai JS, Manolidis S, Barth JL, Stewart MG, Jenkins HA (2000). Unrecognized benign paroxysmal positional vertigo in elderly patients. Otolaryngol Neck Surg.

[20] Motin M, Keren O, Groswasser Z, Gordon CR (2005). Benign paroxysmal positional vertigo as the cause of dizziness in patients after severe traumatic brain injury: diagnosis and treatment. Brain Inj.

[21] Ahn S-K, Jeon S-Y, Kim J-P, Park J, Hur D, Kim D-W (2011). Clinical characteristics and treatment of benign paroxysmal positional vertigo after traumatic brain injury. J Trauma.

[22] Hoffer M, Gottshall K, Moore R, Balough B, Wester D (2004). Characterizing and treating dizziness after mild head trauma. Otol Neurotol.

[23] Smith R, Calzolari E, Seemungal BM (2018). Benign paroxymsal postional vertigo in acute traumatic brain injury [abstract]. J Vestib Res.

[24] Gordon C, Levite R, Joffe V, Gadoth N (2004). Is posttraumatic benign paroxysmal positional vertigo different from the idiopathic form?. Arch Neurol.

[25] Bhattacharyya N (2017). al GSP et. Clinical practice guideline: benign paroxysmal positional vertigo (update). Am Acad Otolaryngol.

[26] Killington MJ, Speck K, Kahlbaum J, Fabian J, Edwards D, Stobie J (2015). Quality-of-life for individuals with a vestibular impairment following an acquired brain injury (ABI); the clients’ perspective. Brain Inj.

[27] Calzolari E, Chepisheva, M, Smith RM, Hellyer P, Tahtis V, Arshad Q, Jolly A, Mahmud M, Wilson M, Rust H, Sharp D, Seemungal BM. Vestibular agnosia in traumatic brain injury and its link to imbalance. Brain. 2020. In Press. 10.1093/brain/awaa386 PMC7880674 33367536

[28] Smith R, Marsden J, Burgess C, Seemungal BM. Why is routine vestibular screening not undertaken by trauma ward staff? A qualitative study. In 2018.

[29] Moran CG, Lecky F, Bouamra O, Lawrence T, Edwards A, Woodford M, et al. Changing the system - major trauma patients and their outcomes in the NHS (England) 2008–17. EClinicalMedicine. 2018;2:13–21. 10.1016/j.eclinm.2018.07.001 PMC6537569 31193723

[30] NICE. Benign paroxysmal positional vertigo: clinical knowledge summaries [Internet]. 2003.

[31] Lacour M, Bernard-Demanze L (2015). Interaction between vestibular compensation mechanisms and vestibular rehabilitation therapy: 10 recommendations for optimal functional recovery. Front Neurol.

[32] Shepard NTTS. Programmatic vestibular rehabilitation. Otolaryngol Head Neck Surg. 1995. 10.1016/S0194-59989570317-9 7816453

[33] Hildebrand M, Host H, Binder E, Carpenter B, Freedland K, Morrow-Howell N (2012). Measuring treatment fidelity in a rehabiliation intervention study. Arch Phys Med Rehabil.

[34] Jacobson GP, Newman CW. The development of the Dizziness Handicap Inventory. Vol. 116, Archives of otolaryngology--head & neck surgery. 1990. 424–427 p. 10.1001/archotol.1990.01870040046011 2317323

[35] Honrubia V, Bell TS, Harris MR, Baloh RW, Fisher LM (1996). Quantitative evaluation of dizziness characteristics and impact on quality of life. Am J Otol.

[36] Lajoie Y, Gallagher SP (2004). Predicting falls within the elderly community: comparison of postural sway, reaction time, the berg balance scale and the activities-specific balance confidence (ABC) scale for comparing fallers and non-fallers. Arch Gerontol Geriatr.

[37] de Guise E, Alturki AY, LeBlanc J, Champoux M-C, Couturier C, Lamoureux J (2014). The Montreal cognitive assessment in persons with traumatic brain injury. Appl Neuropsychol Adult.

[38] Whitehead SJ, Ali S (2010). Health outcomes in economic evaluation: the QALY and utilities. Br Med Bull.

[39] Bjelland I, Dahl AA, Tangen T, Neckelmann D. Bjelland 2002, JoPR, HADS. 2002;52:69–77.

[40] Weir J, Steyerberg EW, Butcher I, Lu J, Lingsma HF, McHugh GS (2012). Does the extended Glasgow outcome scale add value to the conventional Glasgow outcome scale?. J Neurotrauma.

[41] Chu SY, Tsai YH, Xiao SH, Huang SJ, Yang CC (2017). Quality of return to work in patients with mild traumatic brain injury: a prospective investigation of associations among post-concussion symptoms, neuropsychological functions, working status and stability. Brain Inj.

[42] von Steinbüchel N, Wilson L, Gibbons H, Hawthorne G, Höfer S, Schmidt S (2010). Quality of life after brain injury (QOLIBRI): scale development and metric properties. J Neurotrauma.

[43] Horn LB, Rice T, Stoskus JL, Lambert KH, Dannenbaum E, Scherer MR (2015). Measurement characteristics and clinical utility of the clinical test of sensory interaction on balance (CTSIB) and modified CTSIB in individuals with vestibular dysfunction. Arch Phys Med Rehabil.

[44] Matsuda PN, Taylor CS, Shumway-Cook A (2014). Evidence for the validity of the modified dynamic gait index across diagnostic groups. Phys Ther.

[45] Shumway-Cook A, Taylor CS, Matsuda PN, Studer MT, Whetten BK (2013). Modified dynamic gait index (mDGI). Phys Ther.

[46] Sim J, Lewis M (2012). The size of a pilot study for a clinical trial should be calculated in relation to considerations of precision and efficiency. J Clin Epidemiol.

[47] Sacco RR, Burmeister DB, Rupp VA, Greenberg MR (2014). Management of benign paroxysmal positional vertigo: a randomized controlled trial. J Emerg Med.

[48] Amor-Dorado JC, Barreira-Fernandez MP, Aran-Gonzalez I, Casariego-Vales E, Llorca J, Gonzalez-Gay MA (2012). Particle repositioning maneuver versus Brandt-daroff exercise for treatment of unilateral idiopathic BPPV of the posterior semicircular canal: a randomized prospective clinical trial with short- and long-term outcome. Otol Neurotol.

[49] Bernhardt J, Dewey H, Thrift A, Collier J, Donnan G, Early AV, et al. A Very Early Rehabilitation Trial for Stroke (AVERT) Phase II Safety and Feasibility. 2008;390–6. 10.1161/STROKEAHA.107.492363 18174489

[50] Majd S, Apps LD, Hudson N, Hewitt S, Eglinton E, Murphy A (2016). Protocol for a feasibility study to inform the development of a multicentre randomised controlled trial of asthma-tailored pulmonary rehabilitation versus usual care for individuals with severe asthma. BMJ Open.

[51] O’Cathain A, Hoddinott P, Lewin S, Thomas KJ, Young B, Adamson J (2015). Maximising the impact of qualitative research in feasibility studies for randomised controlled trials: guidance for researchers. Pilot Feasibil Stud.

[52] Eldridge SM, Chan CL, Campbell MJ, Bond CM, Hopewell S, Thabane L (2016). CONSORT 2010 statement: extension to randomised pilot and feasibility trials. Pilot Feasibil Stud.

[53] Gale NK, Heath G, Cameron E, Rashid S, Redwood S (2013). Using the framework method for the analysis of qualitative data in multi-disciplinary health research. BMC Med Res Methodol.

[54] Michie S, Johnston M, Abraham C, Lawton R, Parker D, Walker A. Making psychological theory useful for implementing evidence based practice: a consensus approach. Qual Saf Heal Care. 2005 Feb 1;14(1):26 LP – 33. 10.1136/qshc.2004.011155 PMC1743963 15692000

[55] Harris PA, Taylor R, Thielke R, Payne J, Gonzalez N, Conde JG (2009). Research electronic data capture (REDCap)—a metadata-driven methodology and workflow process for providing translational research informatics support. J Biomed Inform.

[56] Harris PA, Taylor R, Minor BL, Elliott V, Fernandez M, O’Neal L, et al. The REDCap consortium: Building an international community of software platform partners. J Biomed Inform. 2019;95:103208. 10.1016/j.jbi.2019.103208 PMC7254481 31078660

[57] Radford K, Phillips J, Drummond A, Sach T, Walker M, Tyerman A (2013). Return to work after traumatic brain injury: cohort comparison and economic evaluation. Brain Inj.

[58] Juengst SB, Adams LM, Bogner JA, Arenth PM, O’Neil-Pirozzi TM, Dreer LE (2015). Trajectories of life satisfaction after TBI: influence of life roles, age, cognitive disability, and depressive symptoms. Rehabil Psychol.

[59] Menon DK. Unique challenges in clinical trials in traumatic brain injury. Crit Care Med. 2009;37(1). 10.1097/CCM.0b013e3181921225 19104212

